# Effect of Thermal Processing on Antioxidant Activity and Cytotoxicity of Waste Potato Juice

**DOI:** 10.1515/biol-2019-0017

**Published:** 2019-05-21

**Authors:** Przemysław Łukasz Kowalczewski, Anna Olejnik, Wojciech Białas, Piotr Kubiak, Aleksander Siger, Marcin Nowicki, Grażyna Lewandowicz

**Affiliations:** 1 Institute of Food Technology of Plant Origin, Poznań University of Life Sciences, 31 Wojska Polskiego Str., 60-624 Poznań, Poland; 2 Department of Biotechnology and Food Microbiology, Poznań University of Life Sciences, 48 Wojska Polskiego Str., 60-627 Poznań, Poland; 3 Department of Biochemistry and Food Analysis, Poznań University of Life Sciences, 48 Mazowiecka Str., 60-623 Poznań, Poland; 4 Department of Entomology and Plant Pathology, Institute of Agriculture, University of Tennessee, 363 Plant Biotechnology Building, 2505 EJ Chapman Drive, Knoxville, TN 37996-4560, USA

**Keywords:** thermal treatment, preservation, by-product, glycoalkaloids content, cancer cells, industrial wastes valorization

## Abstract

Potato juice (PJ), commonly considered a burdensome waste, is rich in various compounds with bioactive properties. It has long been considered a remedy for gastric problems in traditional folk medicine. If valorization of PJ through implementation in the production of functional foods is to be considered, stabilization methods must be developed to allow long-term storage of this seasonal product. It is important that such methods are chosen with regard to their effect on the bioactive value of the obtained product. In this study, the impact of four stabilization methods on the antioxidant and cytotoxic activities of PJ was investigated. Elevated temperatures were used in thermal deproteinization used to obtain DPJW (deproteinated potato juice water) and spray-drying of FPJ (fresh potato juice) that resulted in SDPJ. Freeze drying and cryoconcentration were the low temperature processing methods that yielded PJL (potato juice lyophilisate) and CPJ (cryocorncentrated potato juice), respectively. All processed materials were characterized chemically and compared with raw materials in terms of phenolic compounds content, antioxidant activity as well as cytotoxicity to human tumor cells isolated from the gastric mucosa (Hs476T cell line), colon (Caco-2 and HT-29 cell lines), and normal cells isolated from the small intestine and colon epithelium (IEC-6 and NCM460 cell lines). It was stated that high-temperature processes – thermal deproteinization and spray-drying – yielded products with increased antioxidant potential (TEAC) that also showed increased cytotoxic activity towards intestinal cancer cells. At the same time the cytotoxicity towards normal cells remained on par with that of fresh PJ (IEC-6 cells) or decreased (NCM460 cells). Thermal deproteinization significantly decreased the content of glycoalcaloids in the juice, while spray drying did not have such an effect. The two low-temperature processes investigated – cryoconcentration and freeze drying – did not affect the PJ cytotoxic activity towards any of the cell lines used in the tests, whereas they did affect the antioxidant properties and glycoalcaloids content of PJ.

## 1 Introduction

Potato (*Solanum tuberosum* L.) juice (PJ) is a by-product of the starch industry. Its composition is analogous to potato tuber with the exception of starch, the main product of the starch production process, and fiber [1]. In addition, it is rich in biologically active compounds such as β-carotene, polyphenols, ascorbic acid, tocopherol or α-lipoic acid [2, 3]. PJ has been recognized for its potential for the production of functional food because it contains appreciable quantities of compounds with antioxidant properties. Chlorogenic acid, the most abundant phenolic compound in PJ, constitutes more than 90% of the total content of these compounds [4, 5]. Its high content contributes to the protective role against the development of many chronic diseases. As a part of folk medicine, the use of freshly squeezed potato as a therapeutic agent for the treatment of gastrointestinal complaints goes back to the first decade of the 19^th^ century [6, 7]. The alkalizing and diastolic properties of PJ were believed to be effective in treating stomach ulcers which, however, have not been adequately understood medical problems at that time. *In vitro* and *in vivo* studies on spray-dried PJ (SDPJ) demonstrated its efficacy in inhibiting the inflammatory response and reducing the induced stomach ulcers in rats [8]. In addition, studies indicated that the protease inhibitors present in the potato protein fraction inhibit the fecal proteolytic activity almost completely [9]. Therefore, the use of potato proteins can be a new approach in preventing the‐induced peri‐anal dermatitis. PJ also contains glycoalkaloids, α-chaconine and α-solanine in quantities dependent on genetic and environmental factors as well as the post-harvest storage conditions [10, 11]. Available data indicated that these compounds show anti-proliferative activity towards cancer cells originating from human skin [12], liver [13], prostate and breast [14].

Research reports published in the recent years indicated the possibility of application of PJ, both fresh and dried, in the production of health-promoting food targeted at people suffering from inflammatory bowel disease [15, 16, 17]. The influence of this additive on the structure of the product was dependent on the form in which it was introduced [18]. Moreover, consumer acceptance tests indicated that this ingredient can act as a spice, often positively influencing the taste of a product. PJ is, however, a seasonal product obtained in Poland between August and December, during the starch production season. Moreover, it is prone to microbiological spoilage and constitutes a burdensome waste because of its high load of organic and inorganic ingredients [19], however, it can also be used as a substrate for the production of valuable metabolites by microbiological methods [20, 21, 22]. Thus, developing a method of PJ stabilization that would allow the preservation of its high biological activity is desirable.

Therefore, the overarching goal of this study was to assess the effect of selected treatment methods on the antioxidant and cytotoxic activities of waste PJ obtained as a by-product of potato starch production.

## 2 Materials and Methods

### 2.1 Experimental Materials

Fresh potato juice (FPJ) and deproteinated potato juice water (DPJW) were collected from WPPZ S.A. Potato Industry Company (Poland) as a side streams from the potato starch extraction process.

DPJW is produced industrially by thermal treatment of FPJ at a temperature of 105 to 120 °C and pressures of 10^5^ to 3 × 10^5^ Pa for up to 10 minutes after acidification to pH of approximately 5.0 [23].

Spray-dried potato juice (SDPJ) was obtained by spray-drying of FPJ in the Mobile Minor™ 2000 Spray Dryer (GEA Co., Denmark) using the following conditions: temperatures of 170 °C at the inlet to the drying chamber and 95 °C at the outlet.

Cryoconcetrate of potato juice (CPJ) was obtained using the Pilot Plant Cold Concentration Unit (Freeze Tec, the Netherlands) using the following parameters: cycle time: 40 s; compression time: 10 s; rinse water temperature of the ice bed: 3.5 °C; rinsing pump operation time: 15 s; opening time of the drain valve: 9 s; closing time of the drain valve: 5 s; temperature during the product discharge phase: -3.8 °C. CPJ with an extract concentration of 30.2 °Bx was obtained as a result of the cryoconcentration process of FPJ with an extract content of 6.1 °Bx.

Potato juice lyophilisate (PJL) was obtained by freeze drying using a LMC-1 lyophilizer (Martin Christ Gefriertrocknungsanlagen GmbH, Germany) at a pressure of 0.1 mbar at 20 °C for 24 hours and then dried at 23 °C for 4 hours. Pre-freezing was performed in the low-temperature freezer BM 690 (Froilabo, France).

### 2.2 Chemical analysis of samples

The total nitrogen content was determined by Kjeldahl’s method according to ISO 1871 [24] and was used to calculate protein content by multiplication by the conversion factor of 6.25. The ash content was determined according to ISO 763 [25].

The α-chaconine and α-solanine concentrations were analyzed using a Waters HPLC system (Waters, Milford, MA) consisting of a pump (Waters 600), a photodiode array detector (Waters 2998 PDA), an autosampler (Waters 2707), a column oven (Waters Jetstream 2 Plus), and XBridge C18 column (3.5 μm, 3.0 × 100 mm) (Waters, Milford, MA). The isocratic separation was carried out at with flow rate of 1.0 ml/min. The injection volume was 10 μL while the column temperature was maintained at 70 °C. The mobile phase was a mixture of acetonitrile and 0.1 M KH_2_PO_4_ (20:80 v/v). Detection was carried out at a wavelength of 200 nm. Quantitative determination of glycoalcaloids was carried out by comparing retention times and diode array spectral characteristics with the corresponding standards.

### 2.3 Biological activity

The total phenolic compounds (TPC) content was determined by the Folin-Ciocalteu colorimetric method [26]. The 0.2 N Folin-Ciocalteau reagent (1.15 mL) and 0.16 mL of sample were mixed together, than after 5 minute, 1.15 mL of sodium carbonate (75 g/L) was added and mixed again. After incubation for 60 min at room temperature in the dark, the absorbance of the reaction mixture absorbance was measured at 765 nm (Multiskan GO, Thermo Fisher Scientific, Finland) against a deionized water blank. The results were expressed as an equivalent of chlorogenic acid (CAE) per 1 g of dry matter.

The antioxidant activity was assessed against the ABTS radical (2,2’-azinobis-(3- ethylbenzothiazoline-6-sulfonic acid) [27]. A 7 mM aqueous solution of ABTS with 2.45 mM potassium persulfate in a ratio of 1:0.5 was prepared and incubated in the dark for 12 hours at 20 ˚C. The radical working solution of ABTS radical was prepared immediately before the measurement by diluting with PBS buffer to obtain an absorbance of 0.7±0.02 at 734 nm wavelength. The analysis was carried out by adding 5 mL of working solution to the tube, 50 μL of sample or water (control), shaking and incubating in a water bath for 6 minutes at 30˚C. After incubation the absorbance of reaction mixture was measured at 734 nm (Multiskan GO, Thermo Fisher Scientific, Vantaa, Finland). Results were presented as Trolox equivalent antioxidant capacity (TEAC) per 1 g of dry matter of the examined material.

*In vitro* studies on cytotoxicity of potato juice and its processing products were carried out with the use of human tumor cells isolated from the gastric mucosa (Hs476T line) and colon (Caco-2 and HT-29 lines). The assays were also performed on non-transformed cells isolated from normal small intestine and colon epithelium (IEC-6 and NCM460 cells). The tumor cell lines used, as well as the rat IEC-6 cell line were from the European Collection of Cell Cultures (ECACC), whereas human NCM460 cell line was obtained from Incell Corporation LLC (USA). Cells were grown in DMEM medium (Dulbecco’s Modified Eagle’s Medium, Sigma-Aldrich, Poland) with 1% essential amino acid mixture (Sigma-Aldrich, Poland), 10% fetal bovine serum (FBS, Gibco BRL, USA) and gentamycin at 50 μg / mL (Sigma-Aldrich, Poland). IEC-6 cell cultures were carried out in DMEM medium with 10% addition of FBS, supplemented with bovine insulin (Sigma-Aldrich, Poland) at 0.1 U/mL. NCM460 cells were cultured in M3Base medium (Incell Corporation LLC, USA) supplemented with 10% FBS. All cell cultures were maintained at 37 ˚C in an atmosphere composed of 5% CO_2_ and 95% air.

Cytotoxicity was determined for 24-hour-aged cell cultures, assayed at an initial cell concentration of 2.5 × 10^4^ cells / cm^2^. The cultures were exposed to the analyzed products for 48 hours while maintaining the standard culture conditions. After exposure, the viability and metabolic activity of the cells were determined using the MTT test, which was carried out according to the procedure described by Mosmann [28]. On the basis of the obtained results, the IC_50_ cytotoxic doses were calculated. The IC_50_ dose determines the concentration of the products tested which results in a 50% reduction in cell proliferation and viability.

### 2.4 Statistical analysis

All measurements were repeated in triplicate, unless otherwise stated. One-way analysis of variance (ANOVA) was carried out independently for each dependent variable. Post-hoc Tukey HSD multiple comparison test was used to identify statistically homogeneous subsets at α = 0.05. Statistical analysis was performed with Statistica 13 software (Dell Software Inc., USA).

## 3 Results and discussion

### 3.1 Antioxidant activity

Proper functioning of the human body depends on many factors, including the maintenance of the redox balance. In other words, the reactive oxygen species (ROS), generated in metabolic processes have to be eliminated [29]. Accumulation of a large quantity of ROS results in oxidative stress. ROS take a part in the oxidation of lipids, proteins and even nucleic acids that lead to changes in cells or their death. This may eventually lead to diseases [30]. Oxidative stress can be reduced by providing compounds with antioxidant activity to the body. It was found that plants used for the production of traditional medicines contain many different phytochemical compounds that reduce oxidative stress and can be used in the treatment of chronic diseases [31].

**Figure 1:**
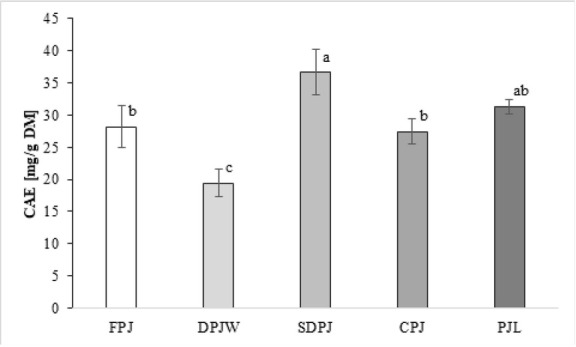
Results of total phenolic compounds content in the analyzed samples Mean values with different letters are significantly different at α=0.05. **CAE** - chlorogenic acid equivalent; **FPJ** – fresh potato juice; **DPJW** - deproteinated potato juice water; **SDPJ** – spray-dried potato juice; **CPJ** – cryoconcetrate of potato juice; **PJL** - potato juice lyophilisate.

In order to stabilize or concentrate it, PJ was processed at high and low temperatures. Elevated temperatures were used in thermal deproteinization used to obtain DPJW and spray-drying of FPJ that resulted in SDPJ. Freeze drying and cryoconcentration were the low temperature processing methods that yielded PJL and CPJ, respectively. The content of total phenolic compounds, expressed as CAE, were different in the analyzed products and the raw material (Fig. 1). Spray-drying of FPJ resulted in an increase in CAE by ca. 30%. The effect of cryoconcentration was statistically insignificant, whereas the thermal deproteinization caused the CAE to decrease by approximately 30%.

**Figure 2:**
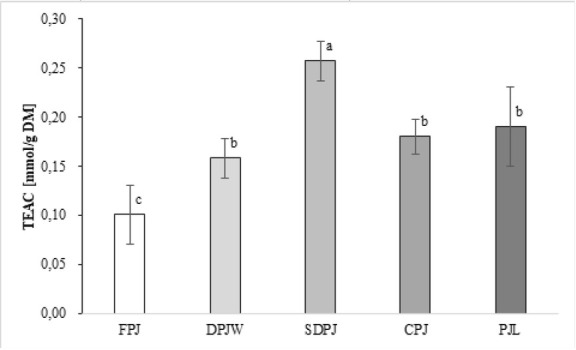
Antioxidant activity of the analyzed PJ treatments. Mean values with different letters are significantly different at α=0.05. **TEAC** - trolox equivalent antioxidant capacity; **FPJ** – fresh potato juice; **DPJW** - deproteinated potato juice water; **SDPJ** – spray-dried potato juice; **CPJ** – cryoconcetrate of potato juice; **PJL** - potato juice lyophilisate.

The observed high value of CAE in the SDPJ was reflected in the results of the antioxidant activity test with ABTS (Fig. 2). The results obtained in the ABTS assay showed that each of the thermal processing methods resulted in increased antioxidant activity of the juice. Similarly to the results of CAE detemination, the best result was observed for SDPJ. The antioxidant activity (ABTS) of FPJ was in accordance to the available data [32, 33]. Increased TEAC values determined for the products of FPJ processing by each method tested were unexpected. In case of the product of spray drying, it seems plausible that the increased antioxidant activity was a result of the formation of new compounds through non-enzymatic browning.

PJ contains both proteins and carbohydrates that form large polymeric melanoidins in Maillard reactions. These compounds are known for their antioxidant properties [34, 35]. Moreover, beside free phenolic acids and polyphenols (chlorogenic acid, catechin, caffeic acid, ferulic acid, gallic acid, and malvidin [36, 37, 38], potato tubers and PJ alike contain bound-form phenolics that are linked to the polysaccharides of cell walls by ester bonds. These compounds can also be formed during thermal treatment of PJ [2, 39].

### 3.2 In vitro cytotoxicity

**Figure 3:**
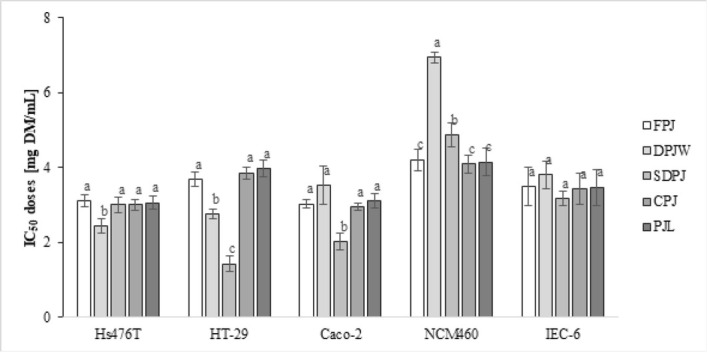
IC_50_ cytotoxic doses [mg DM/mL] calculated for cancerous cells (Caco-2, HT-29 and Hs476T lines) and non-tumorigenic cells (NCM460 and IEC-6 lines). Mean values with different letters are significantly different at α=0.05.

The obtained PJ processing products were subjected to in vitro analyzes of their cytotoxic activity. In Figure 3 the IC_50_ cytotoxic doses of the analyzed products are presented. The observed effect on cell proliferation and viability in model tumor and non-tumor cultures was complex and varied depending on the origin of cell lines. In the case of stomach tumor cells of the Hs476T line, only DPJW showed higher cytotoxic activity than the other analyzed preparations. DPJW cytotoxic dose IC_50_ for Hs476T cells was decreased by approximately 20% (Fig. 3).

In folk medicine, PJ was recommended for problems of the digestive tract, especially those that concerned the intestines [6, 7]. Analyses of the cytotoxicity towards colon cancer cells of the Caco-2 and HT-29 lines showed that both cell lines were the most sensitive to the action of SDPJ. The IC_50_ dose of SDPJ in the case of HT-29 cells was more than twice lower than that of FPJ. In the case of Caco-2 cells it was 33% lower. Significantly higher cytotoxic activity against HT-29 cells was also characteristic for DPJW. Its activity in relation to the Caco-2 line did not differ from the other preparations.

Pharmacological anticancer agents show nonspecific effects. They inhibit the proliferation of both cancerous and normal cells. As a consequence, there is an additional weakening of the organism, which is already struggling with severe disease [40]. The results obtained in this study indicate that PJ preparations are cytotoxic to cells derived from cancer (Caco-2 cells) and normal (NCM460) colon epithelium with different degrees of potency.

Importantly, in the case of the SDPJ and DPJW preparations, their cytotoxicity was specific towards both Caco-2 and HT-29 cancer cell lines (Fig. 3). High cytotoxic activity of the preparations obtained through thermal treatment casts doubt on the biological activity attributed to the protein fraction of the PJ [41, 42]. Our results indicated that low molecular weight compounds resistant to thermal treatment were responsible for that cytotoxic potential rather than the protein fraction. Moreover, the spray-drying process denatures proteins [43, 44], but it can also enhance the therapeutic effect due to the fact that a large portion of active compounds have low affinity for water. Dehydration, even at high temperatures, enhances the activity of these compounds [45].

PJ contains glycoalcaloids, mainly solanine and chaconine, that are present also in the products of its processing (Tab. 1). The acceptable limit for GA content in potatoes is 200 mg / kg of fresh potato tubers [10]. GA are not necessary for the proper growth of the plant, they are secondary metabolites that protect the plant against pests and pathogens. The mechanism of their synthesis is similar to the chlorophyll synthesis plant, hence the belief about the high content of GA in greenery bulbs. It should be noted, however, that although they are processes occurring in similar conditions, they are not directly connected to each other [46]. These compounds are not degraded by the thermal treatment methods commonly utilized in the industry. Glycoalcaloids are regarded as toxic for humans by dietitians and food technologists and, as such, their presence in food is controversial [46, 47]. Excessive consumption of these compounds affects the nervous system by the inhibition of acetylcholinesterase and may cause sweating, vomiting, diarrhea and bronchospasm. Acute poisoning with glycoalcaloids may lead to respiratory and cardiac failures, and coma [48]. Previous studies demonstrated cytotoxic activity of pure glycoalcaloids isolated from potato on colon (Caco-2, HT-29) and liver (HepG2) cancer cells [49, 50, 51]. The thermal deproteinization process resulted in losses in the content of glycoalcaloids (Tab. 1). The amounts of solanine and chaconine were 22% and 51% lower in DPJW then in FPJ. Due to the biological activity demonstrated in this work and documented earlier [8], waste potato juice can thus be used to enrich food, in particular dedicated to people suffering from digestive tract ailments. Published data indicate that it can be successfully used to produce cereal products (pasta [16] and bread [17]), as well as meat products (pâté [18] or sausage [15]). Nonetheless, it is difficult to predict the activity of PJ as it is a complex mixture in which synergistic effects between glycoalcaloids as well as interactions with other of its constituents are possible [52, 53]. Therefore, further metabolomic studies are needed to characterize the compounds present in PJ responsible for biological activity and the mechanisms of these effects observed in current experiments.

**Table 1:** Basic characteristics of fresh and processed potato juice preparations

Parameter [Unit]	FPJ	DPJW	SDPJ	CPJ	PJL
Basic characteristic					
Protein content [g/g]	2.87 ± 0.10^a^	1.41 ± 0.09^b^	2.80 ± 0.12^a^	2.72 ± 0.14^a^	2.81 ± 0.09^a^
Ash content [g/g]	0.91 ± 0.03^b^	1.22 ± 0.06^a^	0.95 ± 0.04^b^	0.96 ± 0.05^b^	0.93 ± 0.04^b^
Glycoalcaloids content					
α-chaconine [μg/g DM]	990.06 ± 17.11^a^	477.12 ± 19.77^b^	982.16 ± 22.31^a^	989.75 ± 14.48^a^	993.02 ± 15.67^a^
α-solanine [μg/g DM]	601.24 ± 19.37^a^	466.18 ± 12.82^b^	603.08 ± 14.12^a^	612.25 ± 14.25^a^	604.31 ± 13.94^a^

Mean values with different letters (a-b) in the rows are significantly different at α=0.05.

## 4 Conclusions

PJ generated during seasonal processing of potatoes in starch production plants, currently considered a burdensome waste, can be valorized into stable products of valuable biological activity. Here, we demonstrated that simple treatment methods allowed the stabilization of PJ and also lead to the improvement of its biological activity.

Thermal treatment of PJ resulted in increased antioxidant potential and positively affected the cytotoxic activity towards intestinal cancer cells (Caco-2 and HT-29). At the same time the cytotoxicity towards normal non-transformed cells remained on par with that of fresh PJ (IEC-6) or decreased (NCM460). Moreover, the content of α-solanine and α-chaconine in the SDPJ was unchanged compared to FPJ, while thermal deproteinization at elevated pressure after acidification (DPJW) caused the content of these glycoalcaloids to decreased significantly. These observations proved a key role of thermostable, rather low-molecular, compounds in biological activity of PJ. However, no correlation to glycoalcaloids content was found.

Low temperature processing yielded products with improved antioxidant activity, unchanged glycoalcaloids content, but no improvement in cytotoxic activity towards cancer cells was observed. As PJ as it is a complex mixture in which synergistic effects between nutrients, vitamins, minerals, and glycoalcaloids are possible, further metabolomic studies are needed to characterize the compounds present in PJ responsible for its biological activity and the mechanisms underlining it.
